# Supplementary figures and images for: Visualizing VDAC1 in live cells using a tetracysteine tag
Source: PLoS One. 2024 Oct 18;19(10):e0311107. doi: 10.1371/journal.pone.0311107 (PMC11488731; doi:10.1371/journal.pone.0311107)

**Figure S1. Mistargeting of VDAC1 is induced by N- and C-terminal fusion of GFP**

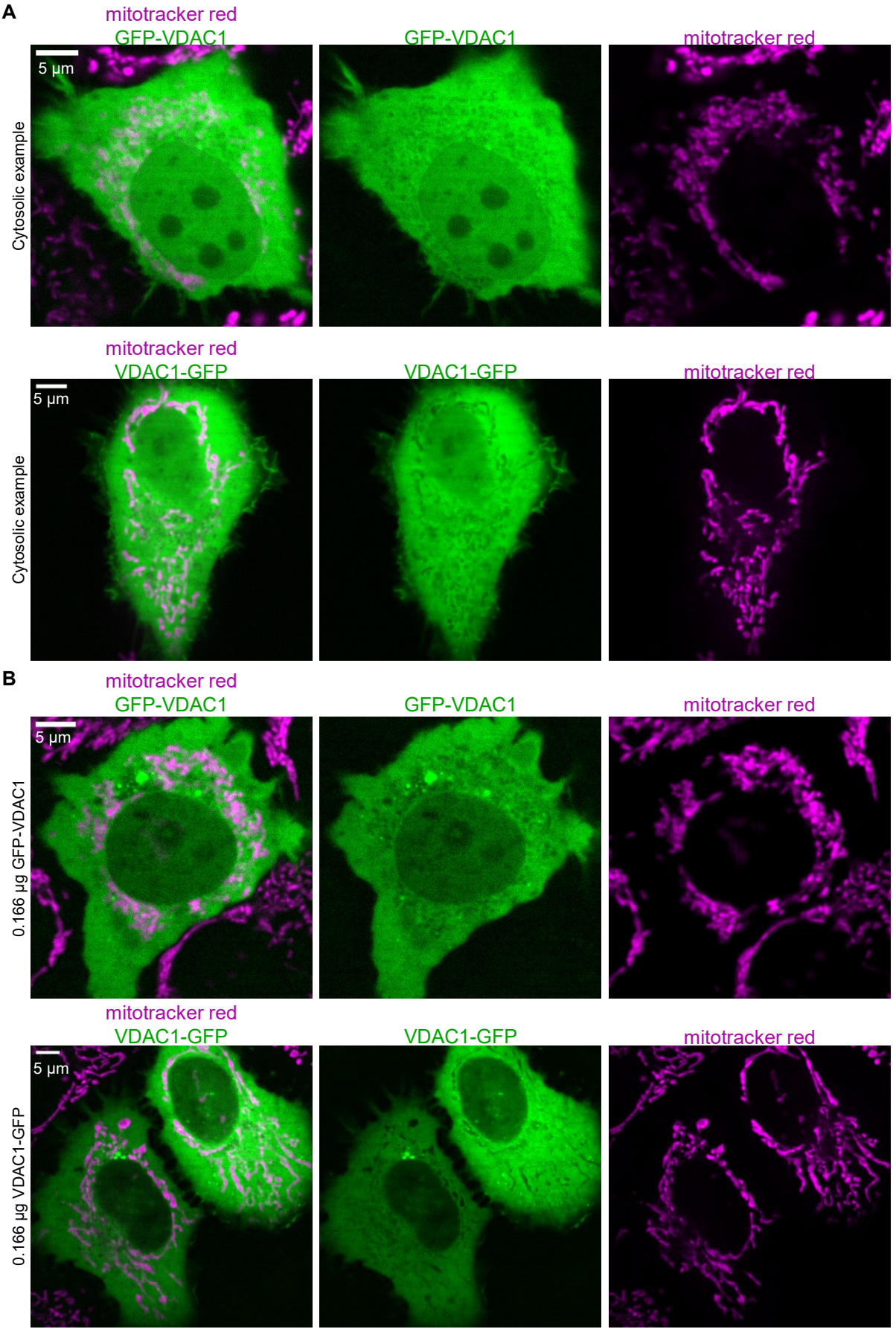

Supplement: S1 Fig — This figure shows examples of HeLa cells expressing FP-tagged VDAC1 without cytosolic aggregation. (PDF) [file pone.0311107.s001.pdf]

**Figure S3. VDAC1-TC-clusters are localized at ER-mitochondria contact sites**

**A**

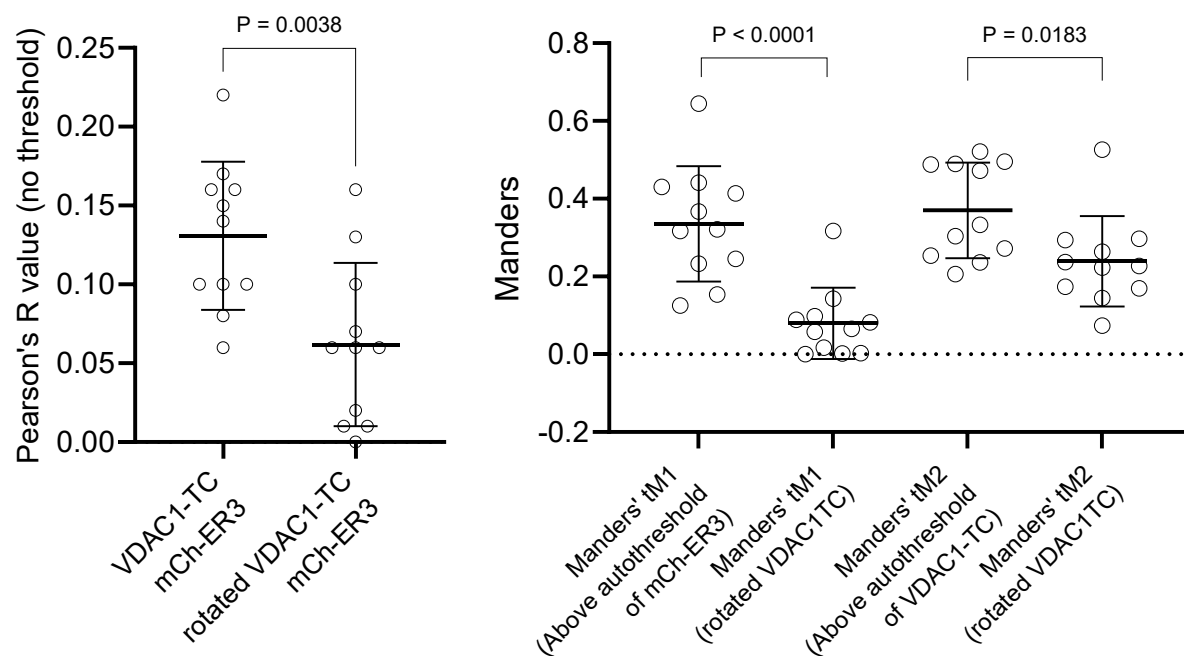

**B**

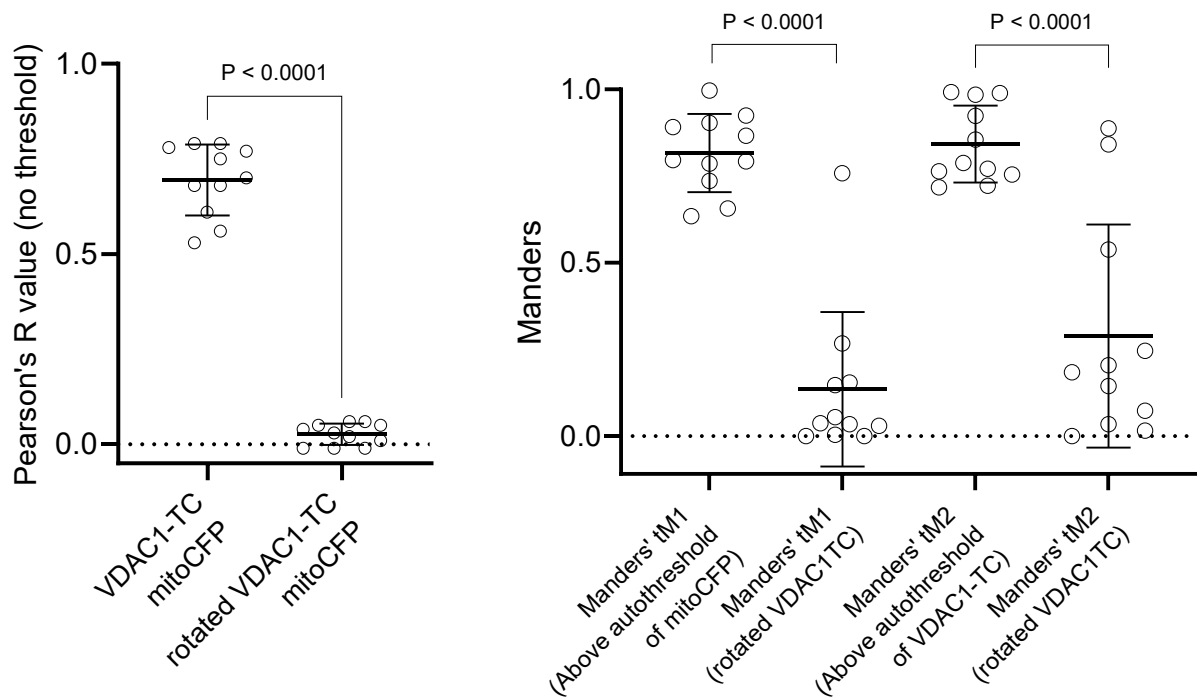

Supplement: S3 Fig — This figure shows Pearson correlation and Manders’ colocalization analysis between the ER, mitochondria and VDAC1-TC. (PDF) [file pone.0311107.s003.pdf]

**Figure S4. VDAC1 colocalizes with BAK-clusters that form in response to stress**

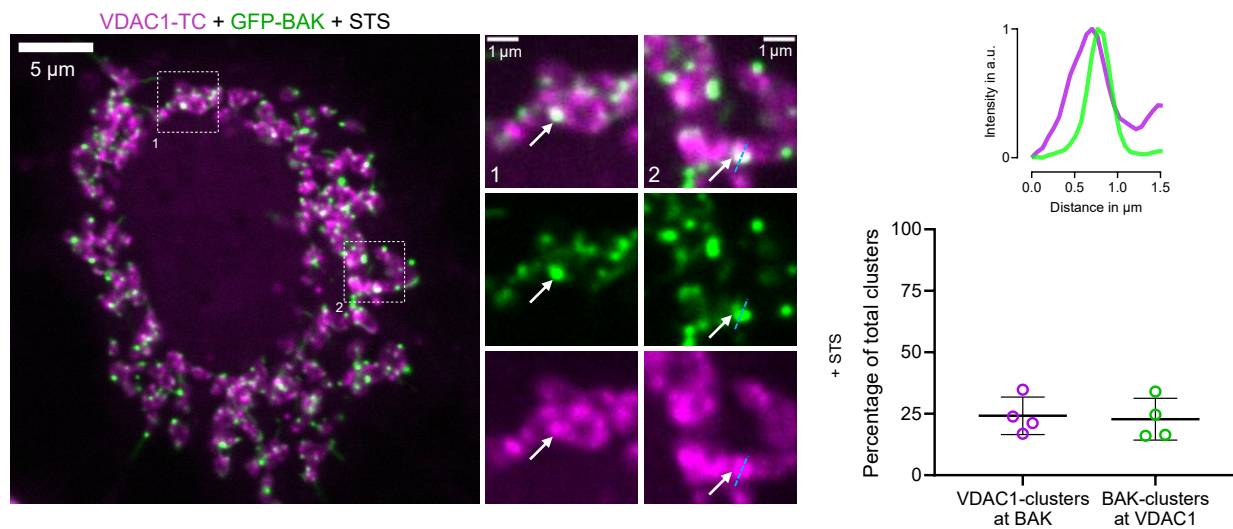

Supplement: S4 Fig — This figure shows confocal images of HeLa cells treated with STS and colocalization analysis between VDAC1-TC and GFP-BAK clusters. (PDF) [file pone.0311107.s004.pdf]

**Figure S5. VDAC1-TC-clusters are observed at mitochondrial fission sites**

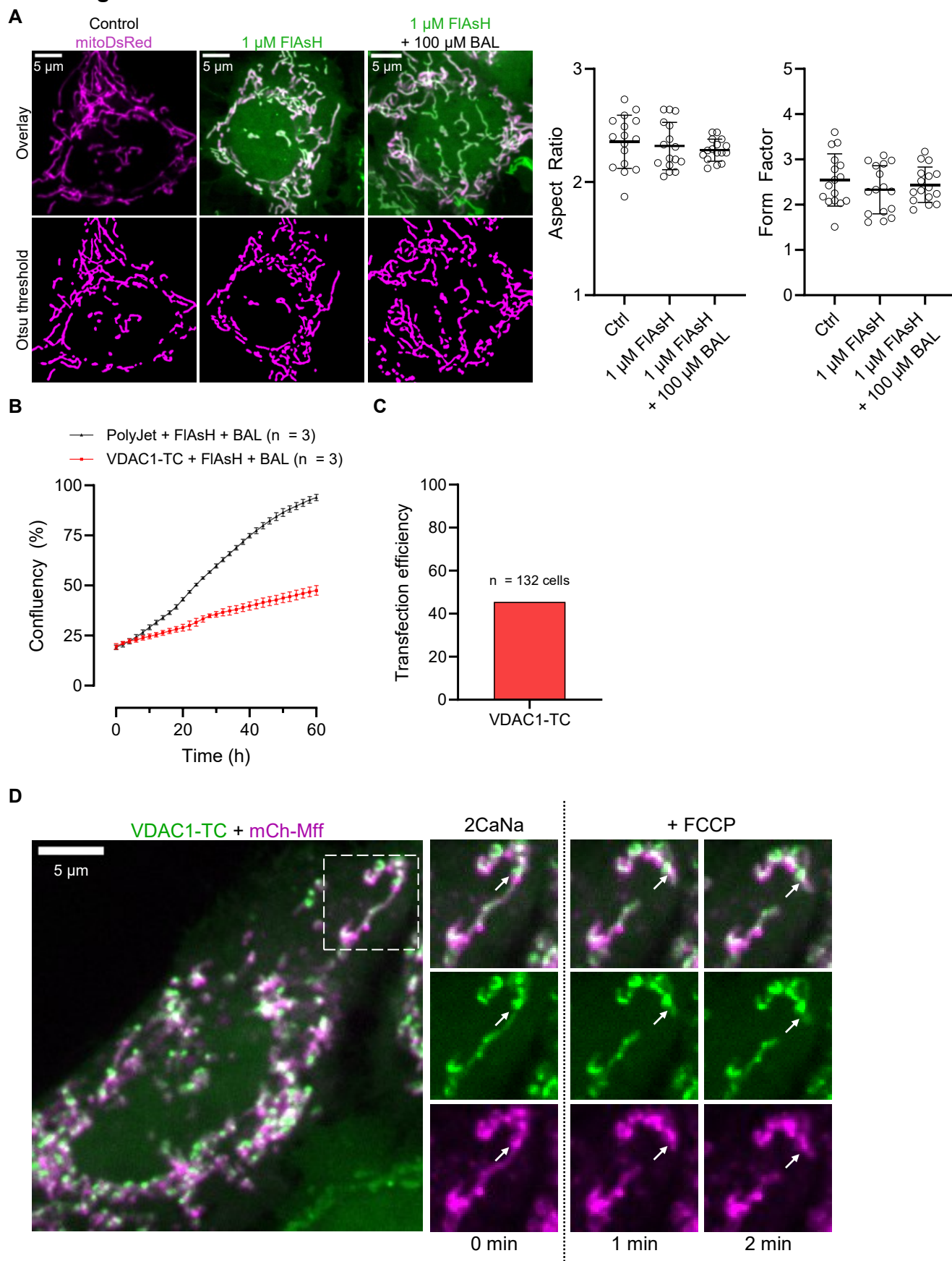

Supplement: S5 Fig — This figure shows analyses of mitochondrial morphology and VDAC1-cluster localization in relation to Mff. (PDF) [file pone.0311107.s005.pdf]
