# Supplementary material for: Visualizing VDAC1 in live cells using a tetracysteine tag
Source: PLoS One. 2024 Oct 18;19(10):e0311107. doi: 10.1371/journal.pone.0311107 (PMC11488731; doi:10.1371/journal.pone.0311107)

Figure S2. Short tetracysteine-tag allows to visualize VDAC1-clusters on mitochondria

A

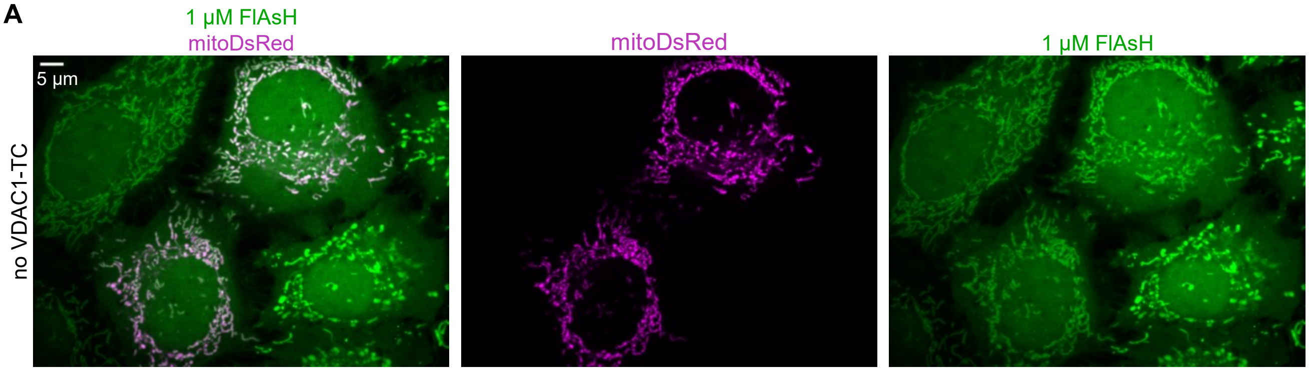

B

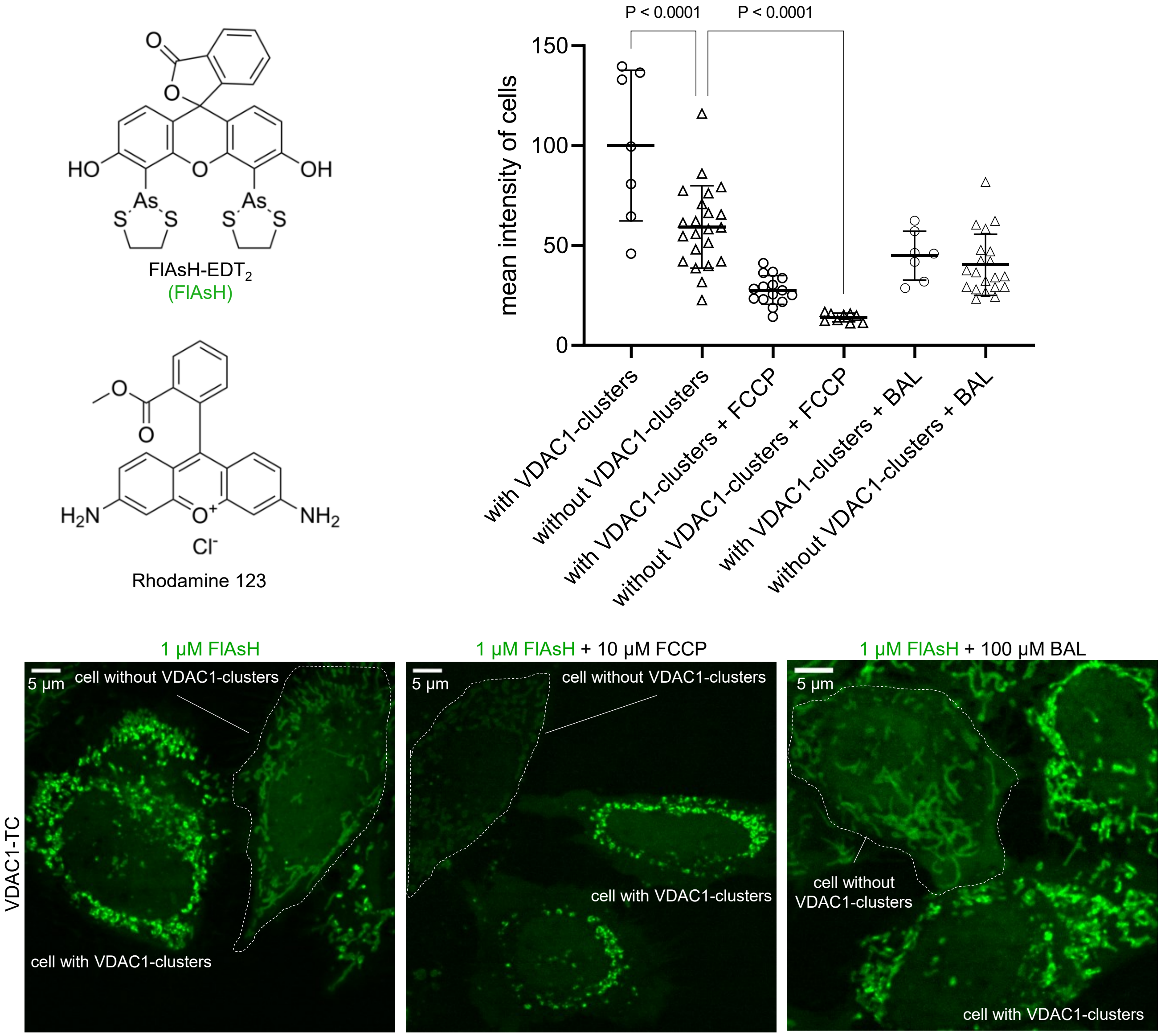

Figure S2. Short tetracysteine-tag allows to visualize VDAC1-clusters on mitochondria

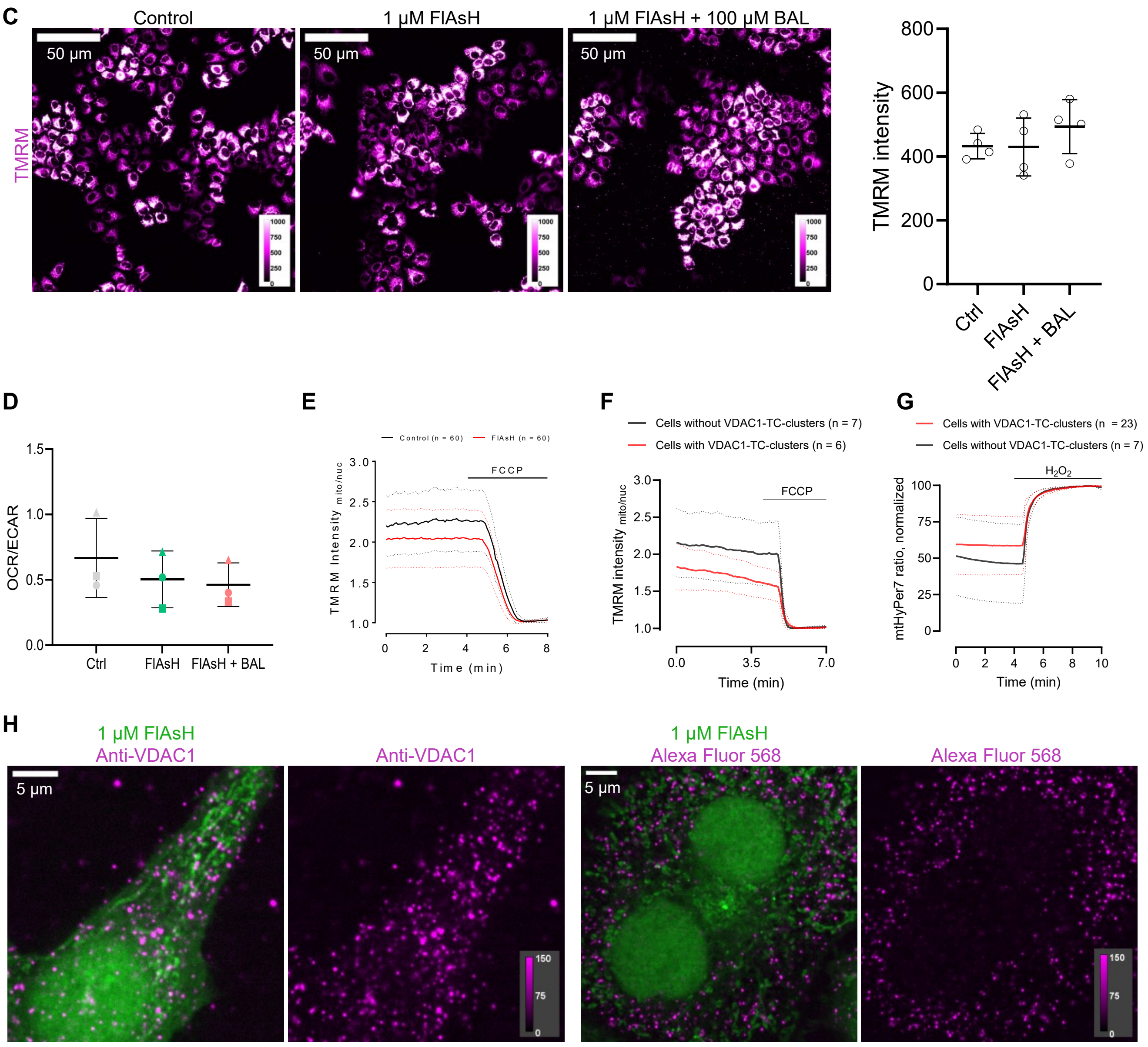

Supplement: S2 Fig — This figure shows confocal images of HeLa cells stained with FlAsH and analyses of mitochondrial function. (PDF) [file pone.0311107.s002.pdf]
