# Supplementary material for: Visualizing VDAC1 in live cells using a tetracysteine tag
Source: PLoS One. 2024 Oct 18;19(10):e0311107. doi: 10.1371/journal.pone.0311107 (PMC11488731; doi:10.1371/journal.pone.0311107)
Supplement: S1 Data — (DOCX) [file pone.0311107.s008.docx]

| Fig. # | Mean | S.D | Statistical method used | P value | # samples |
| --- | --- | --- | --- | --- | --- |
| **Fig. 2B** |  |  |  |  |  |
|  | 0.8744 | 0.03779 | - | - | 9 |
| **Fig. 3A** |  |  |  |  |  |
|  | 63.64 | 8.616 | - | - | 8 |
| **Fig. 4** |  |  |  |  |  |
| VDAC1-clusters at BAK | 37.58 | 5.493 | - | - | 8 |
| BAK-clusters at VDAC1 | 23.12 | 5.437 | - | - | 8 |
| **Fig. S2C** |  |  |  |  |  |
| Ctrl | 432.7 | 39.92 | One-way ANOVA/Tukey | P = 0.4317 | 4 |
| FlAsH | 429.6 | 90.61 |  |  | 4 |
| FlAsH + BAL | 493.7 | 84.60 |  |  | 4 |
| **Fig. S2D** |  |  |  |  |  |
| Ctrl | 0.6672 | 0.3033 | One-way ANOVA/Tukey | P = 0.5639 | 3 |
| FlAsH | 0.5034 | 0.2177 |  |  | 3 |
| FlAsH + BAL | 0.4624 | 0.1670 |  |  | 3 |
| **Fig. S4** |  |  |  |  |  |
| VDAC1-clusters at BAK | 24.19 | 7.639 | - | - | 4 |
| BAK-clusters at VDAC1 | 22.78 | 8.482 | - | - | 4 |
| **Fig. S5A** |  |  |  |  |  |
| AR Ctrl | 2.356 | 0.2351 | One-way ANOVA/Tukey | P = 0.5470 | 16 |
| AR FlAsH | 2.319 | 0.2093 |  |  | 16 |
| AR FlAsH + BAL | 2.282 | 0.09711 |  |  | 16 |
| FF Ctrl | 2.546 | 0.5771 | One-way ANOVA/Tukey | P = 0.4885 | 16 |
| FF FlAsH | 2.330 | 0.5321 |  |  | 16 |
| FF FlAsH + BAL | 2.441 | 0.3913 |  |  | 16 |

| \|  \|  \| \| --- \| --- \| \|  \|  \| |
| --- | --- | --- | --- | --- |
|  |

Minimum data_Pilic et al.. Visualizing VDAC1
