# Supplementary material for: Visualizing VDAC1 in live cells using a tetracysteine tag
Source: PLoS One. 2024 Oct 18;19(10):e0311107. doi: 10.1371/journal.pone.0311107 (PMC11488731; doi:10.1371/journal.pone.0311107)
Supplement: Attachment — Submitted filename: Furkan paper Tetra Cyst ED.pdf [file pone.0311107.s009.pdf]

In this manuscript, the authors addressed the structural organization and localization in sub-cellular compartments of VDAC1 in living cells. To visualization VDAC1 dynamics in living cells, VDAC1 tagged by tetracysteine (TC) and its interaction with CYS-specific fluorogenic reagent were used, allowing its imaging. The results demonstrate that VDAC1 in the mitochondria has a cluster-like organization, and the majority of VDAC1-TC clusters were localized at the MAM. In addition, VDAC1-TC clusters were also identified in mitochondrial fission sites.

This work undoubtedly presents interesting and novel findings on VDAC1 clustering in certain cellular compartments, and points to VDAC1 function in ER–Mito structural and functional cross-talk, as well in mitochondria fission–fusion. However, the following points should be addressed before the manuscript can be accepted for publication.

Comments

1. It has been reported that both GFP-VDAC1 and VDAC1-GFP are predominantly aggregated in the cytosol. It should ne moted, however, that VDAC1-GFP, but not GFP-VDAC1, was found to be targeted to the mitochondria and co-localized with Mito tracker, inducing cell death (VDAC1-GFP at the C-Terminus (see Aram, L., Geula, S., Arbel. N., and Shoshan-Barmatz, V. (2010) VDAC1 cysteine residues: Topology and function in channel activity and apoptosis. *Biochemical J.* **427**, 445-454.).

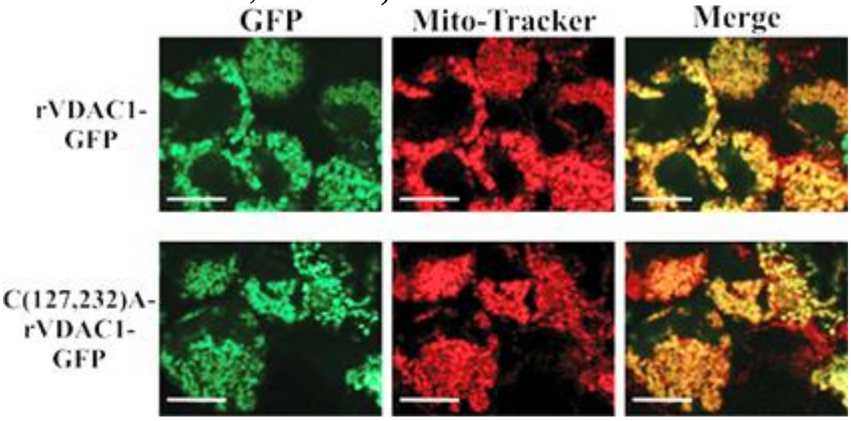

Similarly, even the VDAC1 dimer fused to GFP (VDAC1-VDAC1-GFP) was found to be targeted to the mitochondria (Mader, A., Abu-Hamad, S., Arbel, N., Gutierrez-Aguilar, M., and Shoshan-Barmatz, V. (2010) Dominant-negative VDAC1 mutants reveal oligomeric VDAC1 to be active unit in mitochondria-mediated apoptosis, *Biochemical J.* **429**(1):147-155, Fig. 2).

Aggregation of expressed VDAC1 was observed upon high expression levels. Thus, low plasmid concentration and/or short time transfection may prevent the high production and aggregation.

It is also possible that it is cell-type dependent, as this and other cited studies [3,4] showed poor targeting of C-terminal-tagged VDAC1 in HeLa cells, while the above studies used HEK-293T cells, as well as a different transfection agent and a tetracycline-inducible pcDNA4/TO vector. I suggest indicating that, in other cells and using different transfection agents, VDAC1-GFP can be targeted to the mitochondria and that the mitochondria-targeting sequence is not in either the VDAC1 N- or C-terminus.

2. Using EM, enrichment of VDAC1 in the ER–Mito and Mito–Mito contact sites has been shown in the mouse cerebellum. This more physiological study supports the finding presented here,

and this reviewer suggests indicating it (Shoshan-Barmatz, V., Zalk, R., Gincel, D., and Vardi, N. (2004) Subcellular localization of VDAC in cerebellum and its function in ER-Mitochondria cross-talk. *Biochem. Biophys. Acta* **1657**, 105-114).

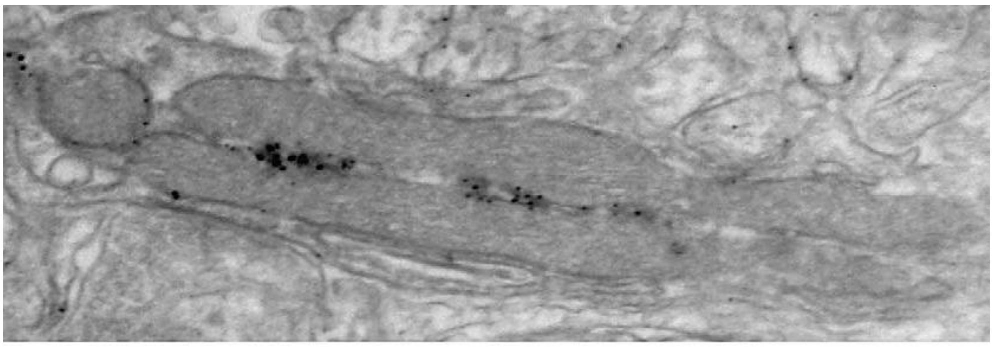

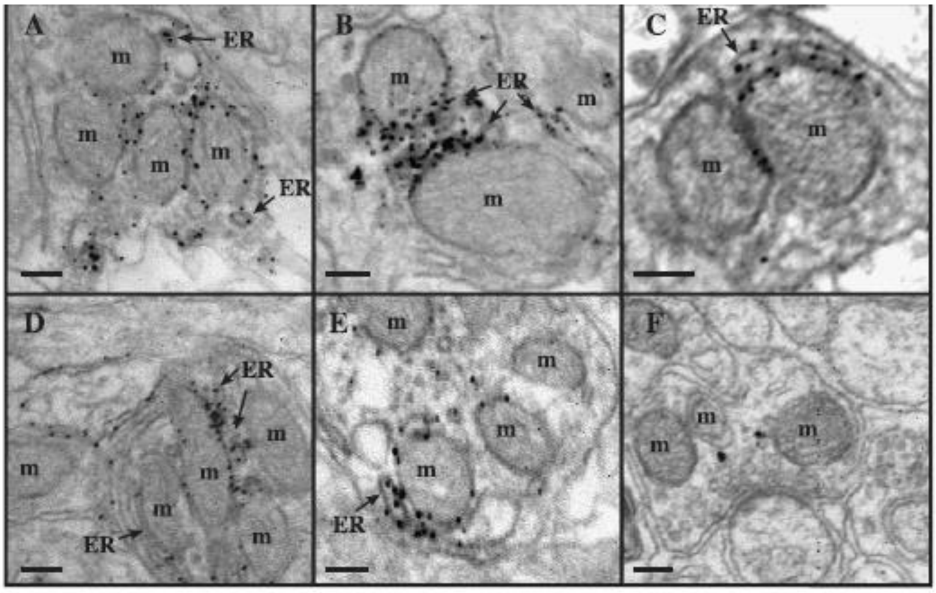

,

3. It will be very interesting to monitor changes in the distribution of VDAC1 clusters upon induction of apoptosis, ER stress, or inhibition of VDAC1 oligomerization. I believe the developed tool of TC-VDAC1 allows this, but it could be the topic of another study.

4. This reviewer could not find any results with Seahorse measurements. Although the method was presented, I may have missed it,

<u>Minor comments:</u>

1. HeLa S3—the meaning of S3 is not well known. Add what it indicates.
2. Line 95—add the composition of the storage buffer
3. Line 149—in the table, the plasmid in which GFP is fused to the C-terminus of VDAC1 is labelled as VDAC1-VDAC1 instead of VDAC1-GFP
4. Concluding sentence—"Since VDAC1-TC is the only tool to image this essential protein in living cells, we believe this approach will provide insights into the role of VDAC1 in cellular pathophysiology"—As indicated in comment 3 above, this tool can also be used under conditions leading to mitochondria dysfunction, apoptosis, autophagy, and more. This is more likely to be used for, rather than in, pathological conditions that are followed more *in vivo*.
